# Gene expression profiling revealed novel mechanism of action of Taxotere and Furtulon in prostate cancer cells

**DOI:** 10.1186/1471-2407-5-7

**Published:** 2005-01-18

**Authors:** Yiwei Li, Maha Hussain, Sarah H Sarkar, James Eliason, Ran Li, Fazlul H Sarkar

**Affiliations:** 1 Departments of Pathology and Internal Medicine, Karmanos Cancer Institute, Wayne State University School of Medicine, Detroit, MI, USA; 2 Division of Hematology/Oncology, Department of Internal Medicine, University of Michigan, Ann Arbor, MI, USA

## Abstract

**Background:**

Both Taxotere and Capecitabine have shown anti-cancer activity against various cancers including prostate cancer. In combination, Taxotere plus Capecitabine has demonstrated higher anti-cancer activity in advanced breast cancers. However, the molecular mechanisms of action of Taxotere and Capecitabine have not been fully elucidated in prostate cancer.

**Methods:**

The total RNA from PC3 and LNCaP prostate cells untreated and treated with 2 nM Taxotere, 110 μM Furtulon (active metabolite of Capecitabine), or 1 nM Taxotere plus 50 μM Furtulon for 6, 36, and 72 hours, was subjected to Affymetrix Human Genome U133A Array analysis. Real-time PCR and Western Blot analysis were conducted to confirm microarray data.

**Results:**

Taxotere and Furtulon down-regulated some genes critical for cell proliferation, cell cycle progression, transcription factor, cell signaling, and oncogenesis, and up-regulated some genes related to the induction of apoptosis, cell cycle arrest, and differentiation in both cell lines. Taxotere and Furtulon also up-regulated some genes responsible for chemotherapeutic resistance, suggesting the induction of cancer cell resistance to these agents.

**Conclusions:**

Taxotere and Furtulon caused the alternation of a large number of genes, many of which may contribute to the molecular mechanisms by which Taxotere and Furtulon inhibit the growth of prostate cancer cells. This information could be utilized for further mechanistic research and for devising optimized therapeutic strategies against prostate cancer.

## Background

Prostate cancer is the most common cancer and the second leading cause of cancer related deaths in men in the United States with an estimated 230,110 new cases and 29,500 deaths in 2004 [1]. Initial treatment for prostate cancer is usually androgen-ablative therapy, radiotherapy or radical prostatectomy and the patients respond to androgen-ablative therapy in the beginning of treatment. However, many patients eventually fail this therapy and die of recurrent androgen-independent prostate cancer and metastasis [2]. Up to 30% of men undergoing radical prostatectomy will also relapse, often as a result of micrometastatic cancer present at the time of surgery [3]. The efficacy of cytotoxic chemotherapy for treatment of hormone-refractory prostate cancer has been tested in clinical trials. In general, response rates of <10% were observed in single-agent studies [2]. Thus, there is a tremendous need for the development of mechanism-based targeted strategies for treatment of prostate cancer.

Taxotere, a member of taxane family, is semi-synthesized from an inactive taxoid precursor extracted from the needles of the European yew, *Taxus baccata*. Its known basic mechanism of action is that it binds to tubulin and deranges the equilibrium between microtubule assembly and disassembly during mitosis [4]. Stabilization of microtubules by Taxotere impairs mitosis and exerts an anticancer effect in tumors [4]. Taxotere has shown clinical activity in wide spectrum of solid tumors including breast, lung, ovarian, prostate cancers, etc [5,6]. In metastatic breast, lung, and ovarian cancer, randomized trials have shown that Taxotere-containing therapies are superior to or as effective as established standard chemotherapeutic regimens and are often associated with an improved safety profile [6]. Clinical trials have also found that weekly Taxotere in patients with metastatic hormone-refractory prostate cancer is associated with improvements in clinical benefit response and quality of life [7,8]. Thus, Taxotere is currently considered to be among the most important anticancer drugs in cancer chemotherapy. In addition to its effects on microtubules, Taxotere also induces apoptosis with down-regulation of bcl_XL _and bcl-2, and upregulation of p21^WAF1 ^and p53 [9,10]. We have previously reported that Taxotere down-regulates some genes for cell proliferation, mitotic spindle formation, transcription factors, and oncogenesis, and up-regulates some genes related to induction of apoptosis and cell cycle arrest in prostate cancer cells, suggesting the pleiotropic effects of Taxotere on prostate cancer cells [11].

Capecitabine is an orally administered systemic prodrug of 5'-deoxy-5-fluorouridine (5-DFUR or Furtulon) which is converted to 5-fluorourasil (5-FU) [12]. Capecitabine is readily absorbed from the gastrointestinal tract. In human and animals, carboxylesterase hydrolyzes much of Capecitabine to 5'-deoxy-5-flurocytidine (5-DFCR). Cytidine deaminase, an enzyme found in most tissues including tumors, subsequently converts 5-DFCR to 5-DFUR. The enzyme, thymidine phosphorylase (dThdPase), then hydrolyzes 5-DFUR to the active drug 5-FU both *in vivo *and *in vitro*. After being converted to 5-FU, Capecitabine inhibits essential cellular biosynthetic processes and is incorporated into DNA to inhibit normal bioprocess function of the cell [13]. Capecitabine has shown anti-tumor activity in various cancers including prostate cancer [14-16]. 5-FU-based chemotherapy improves overall and disease-free survival of patients with cancer. However, response rates for 5-FU-based chemotherapy are low and a large number of tumors eventually becomes resistant to 5-FU [13,17].

Clinical trials showed that chemotherapeutic combination with Taxotere and Capecitabine resulted in improved objective response rate and overall survival without a significant increase in the treatment related adverse effects in metastatic breast cancer and advanced non-small cell lung carcinoma [18,19]. However, the molecular mechanism(s) of action of Taxotere and Capecitabine have not been fully elucidated. In this study, we utilized high-throughput gene chip, which contains 22,215 known genes, to determine the alternation of gene expression profiles of hormone insensitive (PC3) and sensitive (LNCaP) prostate cancer cells exposed to Taxotere and Furtulon. The purpose of this study was: 1) to identify novel genes that have key roles in cancer cell killing and resistance induced by Taxotere and/or Furtulon; 2) to test whether similar genes are altered by Taxotere and Furtulon; 3) to test whether combination therapy alters genes that may reflect better treatment outcome or may provide information whether combination therapy could be antagonistic; 4) finally to provide molecular information for further mechanistic investigation and future clinical application.

## Methods

### Cell culture and growth inhibition

PC3 (ATCC, Manassas, VA) and LNCaP (ATCC) human prostate cancer cells were cultured in RPMI-1640 media (Invitrogen, Carlsbad, CA) supplemented with 10% fetal bovine serum and 1% penicillin and streptomycin in a 5% CO_2_atmosphere at 37°C. Taxotere (Aventis Pharmaceuticals, Bridgewater, NJ) was dissolved in DMSO to make 4 μM stock solution. Furtulon (Roche, Palo Alto, CA) was dissolved in PBS to make 100 mM stock solution. For growth inhibition, PC3 and LNCaP cells were treated with Taxotere (1, 2, and 4 nM), Furtulon (50, 100, and 200 μM), or 1 nM Taxotere plus 50 μM Furtulon for one to three days. Control PC3 and LNCaP cells received 0.01% DMSO or 0.1% PBS for same time points. After treatment, PC3 and LNCaP cells were incubated with MTT (0.5 mg/ml, Sigma, St. Louis, MO) at 37°C for two hours and then with isopropyl alcohol at room temperature for one hour. The spectrophotometric absorbance of the samples was determined by using ULTRA Multifunctional Microplate Reader (TECAN, Durham, NC) at 595 nm. The concentrations of Taxotere and Furtulon used for our *in vitro *studies are easily achievable in humans, suggesting that our experimental results are relevant for human applications. The experiment was repeated three times and a *t *test was performed to verify the significance of cell growth inhibition after treatment.

### Microarray analysis for gene expression profiles

PC3 and LNCaP cells were treated with 2 nM Taxotere, 110 μM Furtulon, or 1 nM Taxotere plus 50 μM Furtulon for 6, 36, and 72 h. Total RNA from each sample was isolated by Trizol (Invitrogen, Carlsbad, CA) and purified by RNeasy Mini Kit and RNase-free DNase Set (QIAGEN, Valencia, CA) according to the manufacturer's protocols. cDNA for each sample was synthesized by Superscript cDNA Synthesis Kit (Invitrogen, Carlsbad, CA) using the T7-(dT)_24 _primer instead of the oligo(dT) provided in the kit. Then, the biotin-labeled cRNA was transcripted *in vitro *from cDNA by using BioArray HighYield RNA Transcript Labeling Kit (ENZO Biochem, New York, NY), and purified by RNeasy Mini Kit. The purified cRNA was fragmented by incubation in fragmentation buffer (40 mM Tris-acetate pH 8.1, 100 mM KOAc, 30 mM MgOAc) at 95°C for 35 min and chilled on ice. The fragmented labeled cRNA was applied to Human Genome U133A Array (Affymetrix, Santa Clara, CA), which contains 22,215 human gene probes, and hybridized to the probes in the array. After washing and staining, the arrays were scanned. Two independent experiments were performed to verify the reproducibility of results.

### Microarray data normalization and analysis

The gene expression levels of samples were normalized and analyzed by using Microarray Suite, MicroDB™, and Data Mining Tool software (Affymetrix, Santa Clara, CA). The absolute call (present, marginal, absent) and average difference of 22,215 gene expressions in a sample, and the absolute call difference, fold change, average difference of gene expressions between two or several samples were normalized and identified using these software. Statistical analysis of the mean expression average difference of genes, which show >2 fold change, was performed using a *t *test between treated and untreated samples. Clustering and annotation of the gene expression were analyzed by using Cluster and TreeView [20], Onto-Express [21], and GenMAPP [22]. Genes that were not annotated or not easily classified were excluded from the functional clustering analysis.

### Real-time RT-PCR analysis for gene expression

To verify the alterations of gene expression at the mRNA level, which appeared on the microarray, we chose representative genes (Table 1) with varying expression profiles for real-time RT-PCR analysis. Two micrograms of total RNA from each sample were subjected to reverse transcription using the Superscript first strand cDNA synthesis kit (Invitrogen, Carlsbad, CA) according to the manufacturer's protocol. Real-time PCR reactions were then carried out in a total of 25 μL reaction mixture (2 μl of cDNA, 12.5 μl of 2X SYBR Green PCR Master Mix, 1.5 μl of each 5 μM forward and reverse primers, and 7.5 μl of H_2_O) in SmartCycler II (Cepheid, Sunnyvale, CA). The PCR program was initiated by 10 min at 95°C before 40 thermal cycles, each of 15 s at 95°C and 1 min at 60°C. Data were analyzed according to the comparative Ct method and were normalized by actin expression in each sample. Melting curves for each PCR reaction were generated to ensure the purity of the amplification product.

### Western blot analysis

We also conducted Western Blot analysis to verify the alterations of genes at the level of translation for selected genes with varying expression profiles. The PC3 and LNCaP cells were treated with 1 and 2 nM Taxotere or 50 and 110 μM Furtulon for 24, 48, and 72 hours. After treatment, the cells were lysed in 62.5 mM Tris-HCl and 2% SDS, and protein concentration was measured using BCA protein assay (PIERCE, Rockford, IL). The proteins were subjected to 10% or 14% SDS-PAGE, and electrophoretically transferred to nitrocellulose membrane. The membranes were incubated with anti-cathepsin C (1:200, Santa Cruz, Santa Cruz, CA), anti-p16 (1:200, Santa Cruz, Santa Cruz, CA), anti-IKKα (1:100, Santa Cruz, Santa Cruz, CA), anti-p21^WAF1 ^(1:500, Upstate, Lake Placid, NY), anti-Bax (1:10000, Trevigen, Gaithersburg, MD), anti-survivin (1:200, Alpha Diagnostic, San Antonio, TX), anti-CDC2 (1:200, Santa Cruz, Santa Cruz, CA), anti-cyclin A (1:250, NeoMarkers, Union City, CA), anti-cyclin B (1:200, Santa Cruz, Santa Cruz, CA), anti-cyclin E (1:250, NeoMarkers), and anti-β-actin (1:10000, Sigma, MO) primary antibodies, and subsequently incubated with secondary antibody conjugated with fluorescence dye. The signal was then detected and quantified by using Odyssey infrared imaging system (LI-COR, Lincoln, NE).

## Results

### Cell growth inhibition

**Figure 1:**
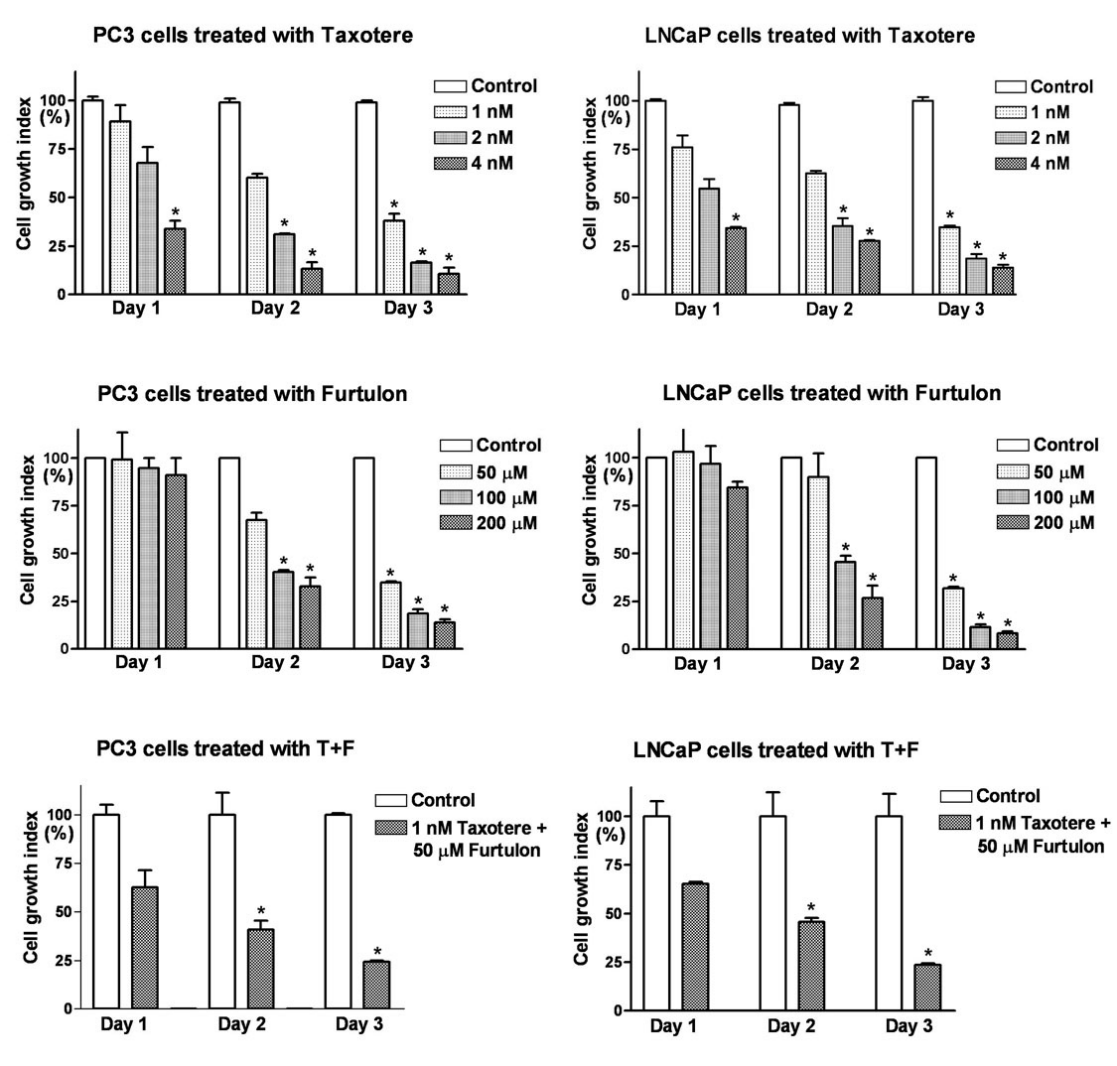
Effects of Taxotere and/or Furtulon on the growth of PC3 and LNCaP Cells. PC3 and LNCaP prostate cancer cells treated with Taxotere and/or Furtulon resulted in a dose and time-dependent inhibition of cell proliferation (* : *p *< 0.05, n = 3)

MTT assay showed that the treatment of PC3 and LNCaP prostate cancer cells with Taxotere, Furtulon, or lower concentration of Taxotere plus Furtulon resulted in dose and time-dependent inhibition of cell proliferation (Figure 1), demonstrating the inhibitory effect of Taxotere and Furtulon on the growth of PC3 and LNCaP prostate cancer cells.

### Regulation of mRNA expression by Taxotere and Furtulon treatment

Microarray analysis showed that the alterations of gene expression were occurred as early as 6 hours of Taxotere and/or Furtulon treatment, and were more evident with longer treatment (Table 2 and 3).

Clustering analysis based on gene function showed down-regulation of some genes for cell proliferation and cell cycle progression (cyclin A, cyclin F, CDC2, CDK2, etc), transcription factors (transcription factor A, ATF5, TAF1131L, FOXM1, etc), and oncogenesis (GRO oncogene, BRCA1 associated RING domain, tumor-associated nuclear protein p120, etc) in Taxotere and/or Furtulon treated prostate cancer cells (Table 2 and 3). In contrast, Taxotere and/or Furtulon up-regulated some genes that are related to the induction of apoptosis (GADD45A, GADD45B, etc), cell cycle arrest (p21^CIP1^, VDUP1, BTG, etc), and tumor suppression (Table 2 and 3).

Combination treatment with Taxotere and Furtulon also altered expression of some genes (CDC27, CDK9, p18, IKKα, etc) that showed no change in mono-treatment (Table 4 and 5), suggesting the synergic effects of combination treatment on some genes.

Taxotere and Furtulon also up-regulated some genes (S-100P, ALDH1A3, casein kinase, annexin, etc) responsible for chemotherapeutic resistance, suggesting the induction of cancer cell resistance to these agents (Table 2 and 3). Taxotere and Furtulon also showed differential effects on PC3 cells with alteration of metastasis-related genes and on LNCaP cells with down-regulation of survivin, cyclin B & E, CDC2, CDC25, and specifically AR by Furtulon, suggesting their effects mediated by both AR-independent and dependent pathways (Table 2 and 3).

### Target verification by real-time RT-PCR and western blot

**Figure 2:**
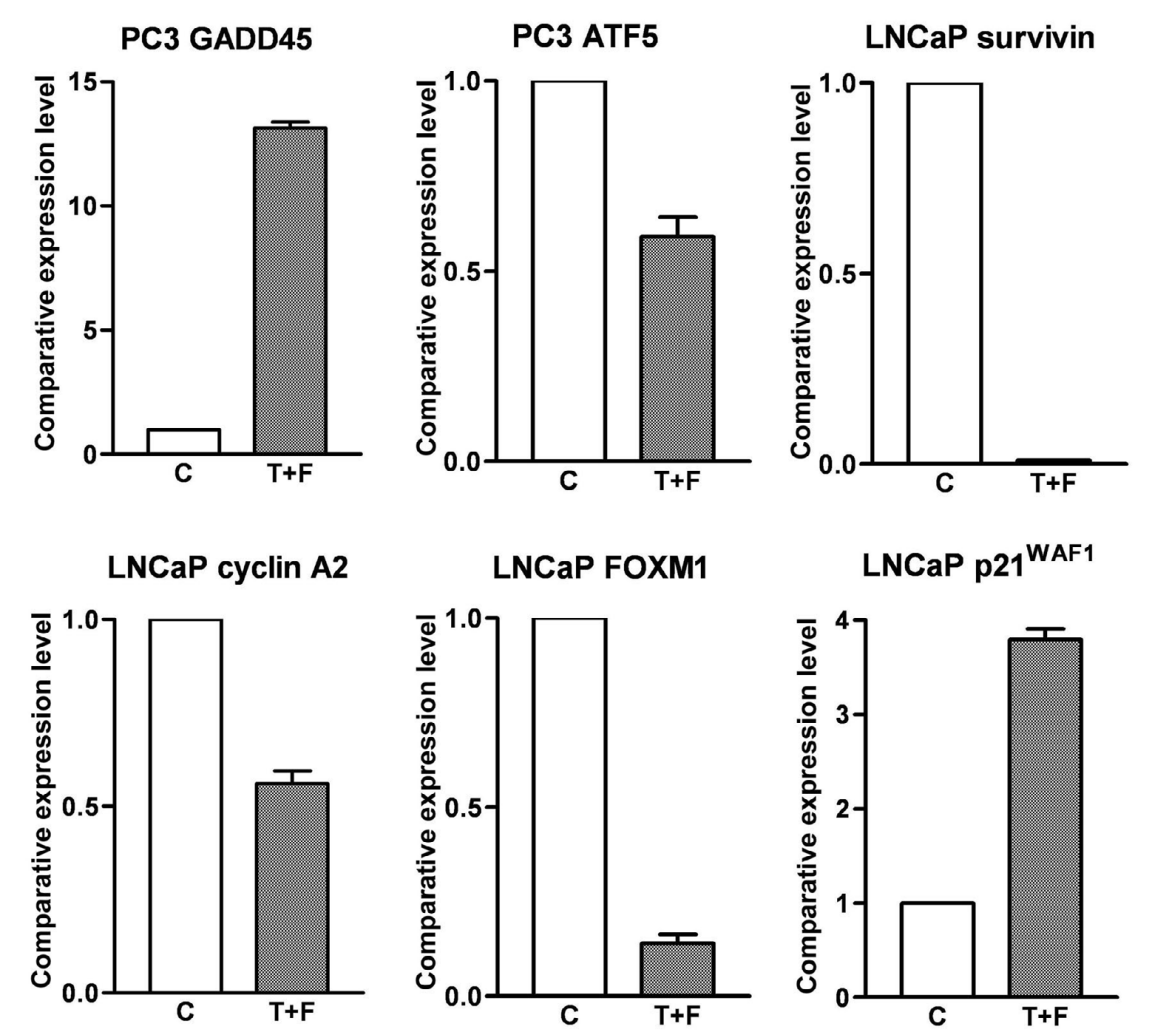
Real-time RT-PCR showing the altered expression of specific genes in Taxotere and Furtulon treated PC3 and LNCaP cells. (C: control; T+F: Taxotere and Furtulon combination treatment.)

**Figure 3:**
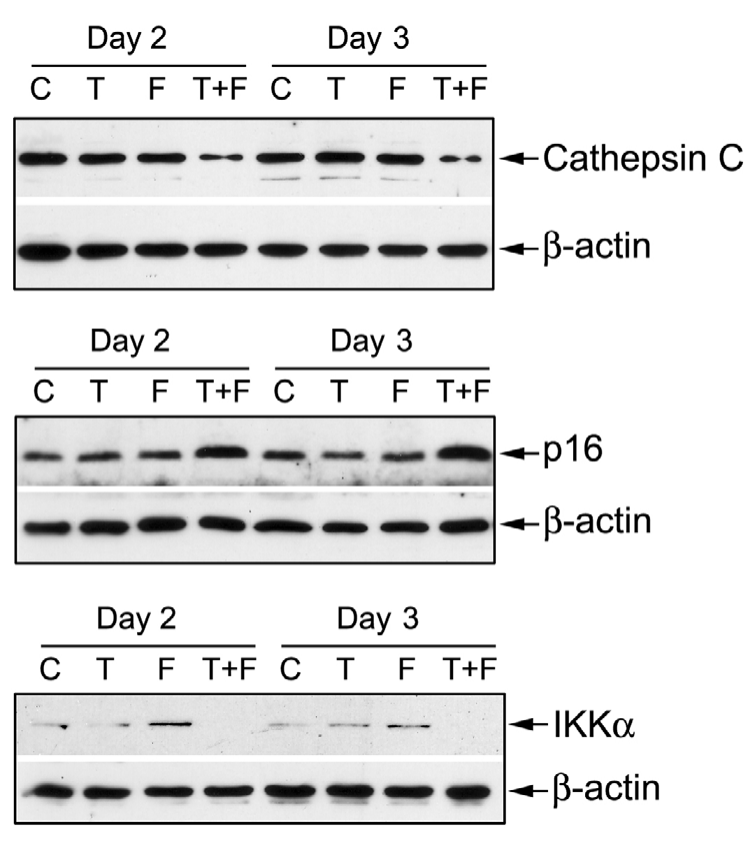
Western Blot analysis showing the altered expression of specific genes in Taxotere and Furtulon treated PC-3 or LNCaP cells. (C: control; T: Taxotere treatment; F: Furtulon treatment; T+F: Taxotere and Furtulon combination treatment.)

To verify the alterations of gene expression at the mRNA level, which appeared on the microarray, we chose representative genes with varying expression profiles for real-time RT-PCR and Western Blot analysis. The results of real-time RT-PCR for these selected genes were in direct agreement with the microarray data (Figure 2). The same alternations of gene expression were observed by real-time RT-PCR analysis, although the fold change in the expression level was not exactly same between these two different analytical methods. The results of Western Blot analysis were also in direct agreement with the microarray and real-time RT-PCR data (Figure 3 and our earlier report [11]). These results support the findings obtained from microarray experiments.

## Discussion

**Figure 4:**
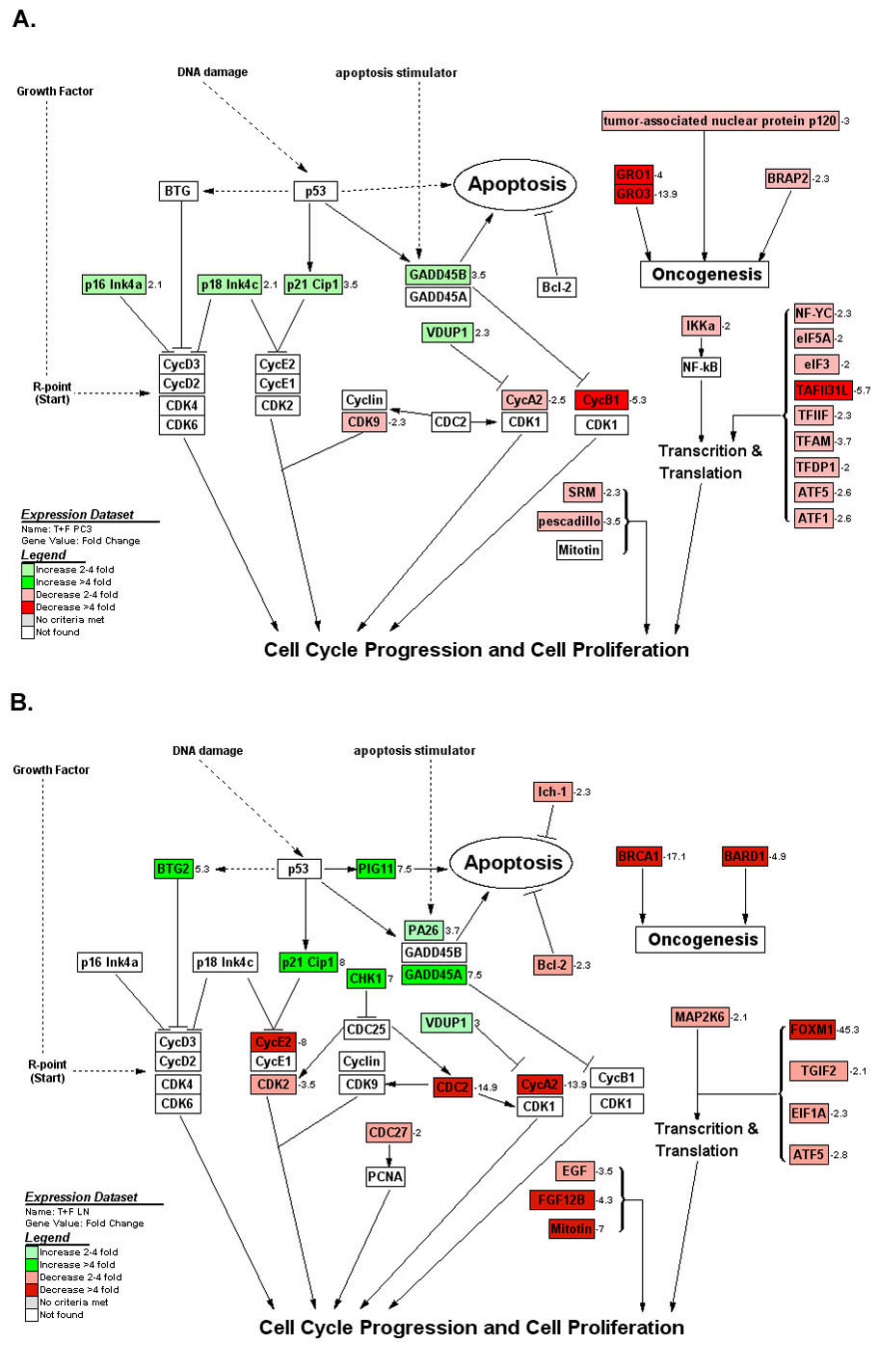
Effects of Taxotere and Furtulon on cell cycle, apoptosis, and other pathway related gene expression analyzed and visualized by GenMAPP software integrated with cDNA microarray data. A: PC3 cells. B: LNCaP cells. (positive value: increase in fold change; negative value: decrease in fold change; A: PC3 cells; B: LNCaP cells)

It has been known that Taxotere binds to microtubules while Capecitabine is incorporated into DNA, inhibiting the bioprocess in cancer cells [4,13]. However, the precise molecular mechanisms for inhibiting cancer cell growth by Taxotere and/or Capecitabine have not been fully elucidated. From gene expression profiles of Taxotere and/or Capecitabine treated prostate cancer cells, we found that these chemotherapeutic agents caused alterations in the expression of many genes related to the control of cell proliferation, apoptosis, transcription, translation, cell signaling, oncogenesis, and angiogenesis (Figure 4), although the cellular target of Taxotere or Capecitabine appears to be different.

It has been well known that CDCs regulate the molecules related to the cell cycle initiation and progression and that cyclins associate with cyclin-dependent protein kinases (CDKs) and CDCs to control the process of cell cycle [23,24]. The CDK inhibitors including p21^WAF1^, p16^INK4A^, and p18^INK4C ^have been demonstrated to arrest the cell cycle and inhibit the growth of cancer cells [23,24]. Our results showed that Cyclins (cyclin A2, cyclin E2, cyclin F, cyclin B1), CDK2, CDC2, and other cell growth promotion genes (pescadillo, spermidine synthase, mitotin) [25-27] were down-regulated in Taxotere and/or Furtulon treated prostate cancer cells, while CDK inhibitor p21^WAF1 ^and other growth inhibitor genes (BTG2, VDUP1, anti-proliferative B-cell translocation gene 1) [28,29] were up-regulated, suggesting that Taxotere and/or Furtulon inhibited the growth of prostate cancer cells through the arrest of cell cycle and the inhibition of cell proliferation (Figure 4). The down-regulation of CDC27, CDK9, EGF, and FGF12B, and up-regulation of p16^INK4A ^and p18^INK4C ^were also observed in combination treatment but not in mono-treatment, suggesting the synergic effect of combination treatment. These observations are novel in Taxotere and/or Furtulon treated prostate cancer cells.

Induction of apoptosis by chemotherapeutic agents also leads to the inhibition of cancer cell growth. It has been reported that Taxotere is able to induce apoptosis by caspase-3 dependent or independent cell death mechanism [30]. Capecitabine may induce apoptosis through Fas/FasL or Bax/Bcl-2 pathway [31,32]. From gene expression profile, we found that Taxotere and/or Furtulon increased level of growth arrest and DNA-damage-inducible alpha (GADD45A), GADD45B, p53 regulated PA26 nuclear protein (PA26), and p53-induced protein 11 (PIG11), all of which are related to the induction of apoptotic processes. GADD45A and GADD45B have been known to promote apoptosis and regulate G2/M arrest [33]. PA26 is a target of the p53 tumor suppressor and a member of the GADD family with the properties of inducing apoptosis [34]. PIG11 as a downstream target of p53 is also involved in the apoptotic processes [35]. The combination treatment also showed down-regulation of negative regulator of programmed cell death ICH-1S and Bcl-2-associated transcription factor, which was not occurred in mono-treatment. The induction of apoptosis mediated by GADD45A, GADD45B, PA25, and PIG11 could be another molecular mechanism by which Taxotere and/or Furtulon inhibit the growth of prostate cancer cells.

We also found that Taxotere and/or Furtulon inhibited the expression of transcription factors (FOXM1, ATF5, TFAM, TAFII31L), translation factors (EIF1A, EIF5A), oncogene (GRO1, GRO3, BRCA1-associated protein, tumor-associated nuclear protein p120), and heat shock protein, and up-regulated the genes for differentiation (prostate differentiation factor). These results are novel, and suggest the beneficial effects of Taxotere and/or Furtulon on the inhibition of cancer cell growth and oncogenesis.

It is important to note that Taxotere and/or Furtulon also up-regulated the expression of some genes which are known to induce cell resistance to chemotherapeutic agents and to favor cell survival. Among these genes, calcium-binding protein S100P has been found to be highly expressed in cells which develop acquired resistance to anti-tumor agents [36]. The overexpression of aldehyde dehydrogenase 1 (ALDH1) has also been detected solely in classical multidrug resistance cancer cells [37,38]. It has been reported that Annexin-I, casein kinase 1, and cisplatin-resistance associated protein expressions modulate drug resistance in tumor cells [39,40]. The up-regulation of these molecules by Taxotere and/or Furtulon could induce cell resistance to chemotherapeutic agents. Also, Taxotere and/or Furtulon were found to up-regulate the expression of Notch 3, angiopoietin, activating transcription factor 3, which could favor cell survival [41-43]. Further in depth mechanistic studies are needed to address these issues. The investigation on overcoming these unbeneficial effects with other agents must be devised, which is ongoing in our laboratory.

Taxotere showed no effect on AR expression while Furtulon down-regulated AR expression in LNCaP cells, suggesting that the combination could be superior in AR-positive cells. The genes altered by Taxotere and/or Furtulon with respect to the control of cell growth, apoptosis, transcription, oncogenesis, and metastasis in androgen insensitive PC3 cells are different from that in androgen sensitive LNCaP cells, suggesting that the effects of Taxotere and Furtulon may be mediated by both AR-dependent and independent signaling pathways. We observed up-regulation of tissue inhibitor of metalloproteinase 1 (TIMP1), TIMP2, and protease inhibitor 3 in Taxotere and/or Furtulon treated PC3 cells, suggesting that Taxotere and/or Furtulon may exert anti-metastatic effect. However, we also observed increase in the expression of MMP1, MMP9, cathepsin B, uPA, and tPA in Taxotere and Furtulon treated PC3 cells, therefore, more experimental studies are needed to reveal the overall effect of Taxotere and Furtulon on metastatic processes. These results were not observed in androgen sensitive LNCaP cells, suggesting difference in effects that could be mediated through different cell signal transduction pathways.

## Conclusions

In conclusion, Taxotere and/or Furtulon directly and indirectly caused changes in the expression of many genes that are critically involved in the control of cell proliferation, apoptosis, transcription, translation, oncogenesis, angiogenesis, metastasis, and drug resistance (Figure 4). These findings could provide molecular information for further investigation on the mechanisms by which Taxotere and Furtulon exerts their pleiotropic effects on prostate cancer cells. These results could also be important in devising mechanism-based targeted therapeutic strategies for prostate cancer, especially in devising combination therapy for drug resistant prostate cancers. However, further in-depth investigations are needed in order to establish cause and effect relationships between these altered genes and therapeutic response in prostate cancer cells.

## Competing interests

The author(s) declare that they have no competing interests.

## Authors' contributions

FHS designed the study and prepared the manuscript. YL carried out cell growth inhibition, microarray and Western Blot analysis and drafted the manuscript. MH participated in the design of the study. RL and SHS carried out real-time PCR. JE prepared Furtulon reagent. All authors read and approved the manuscript.

## Pre-publication history

The pre-publication history for this paper can be accessed here:



## Figures and Tables

**Table 1:** The primers used for real-time RT-PCR analysis

**Genes**	**Primer Sequence**	**PCR Product**
ATF5	ctc ctc ctt ctc cac ctc aa gcc gac ttg ttc tgg tct ct	103 bp
Cyclin A2	aat cag ttt ctt acc caa tac ctg atg gca aat act tga	127 bp
Fas/Apo-1	caa aag tgt taa tgc cca agt gca gtc tgg ttc atc cc	187 bp
FOXM1	gcc aca ctt agc gag acc c atc aca agc att tcc gag aca	189 bp
GADD45	cgc ctg tga gtg agt gc ctt atc cat cct ttc ggt ctt	154 bp
IGFBP2	atg ggc gag ggc act t cag ctc ctt cat acc cga ctt	189 bp
uPA	ggg agc aga gac act aac gac t ctc aca gcc cac aca aca ag	108 bp
Ki-67	ccg ggc tcc atc atc t ctc cag acg cca aaa taa gac	148 bp
p21^WAF1^	ctg gag act ctc agg gtc gaa gga tta ggg ctt cct ctt gga	98 bp
p27^KIP1^	cgc tcg cca gtc cat t aca aaa ccg aac aaa aca aag	187 bp
PIR	cac tag ccc tcc atc ctc tac ggg tct gcc aat gct tct	151 bp
MMP1	gct ttc ctc cac tgc tgc t aac ttg cct ccc atc att ctt	146 bp
STK6	tca gcg ggt ctt gtg t ctc ttt tgg gtg tta ttc agt	162 bp
Survivin	cca ctg ccc cac tga gaa c acc gga cga atg ctt ttt atg	118 bp
TRIP13	tct ggc agt gga caa gca gtt tgg gag acg gct gtg tgg	136 bp
GAPDH	ctg cac cac caa ctg ctt ag ttc agc tca ggg atg acc tt	222 bp
β-actin	cca cac tgt gcc cat cta cg agg atc ttc atg agg tag tca gtc ag	99 bp

**Table 2:** Fold change of genes in PC3 cells exposed to combination treatment or mono-treatment with Taxotere or Furtulon

**Gene**	**Taxotere**			**Furtulon**			**Taxotere+Furtulon**		
			
	**6 h**	**36 h**	**72 h**	**6 h**	**36 h**	**72 h**	**6 h**	**36 h**	**72 h**
***Cell cycle and apoptosis***									
NM_001237.1 cyclin A2 (CCNA2)	NC	-1.1	-2.0	-1.1	-1.7	-3.0	-1.1	-1.4	-2.5
BE407516 cyclin B1	-1.1	-1.1	-2.3	NC	-3.0	-6.1	-1.1	-3.0	-5.3
NM_001761.1 cyclin F (CCNF)	-1.3	-1.3	-2.5	-1.5	-2.5	-2.6	-1.6	-1.6	-2.5
NM_014303.1 pescadillo homolog 1	NC	-1.5	-2.3	1.3	-1.9	-2.6	-1.1	-2.1	-3.5
NM_003132.1 spermidine synthase (SRM)	NC	-1.5	-2.5	-1.1	-1.3	-2.5	-1.1	-1.1	-2.3
NM_000389.1 cyclin-dependent kinase inhibitor 1A (p21, Cip1)	1.7	1.0	2.6	1.9	2.6	3.5	NC	1.7	3.5
NM_003914.1 cyclin A1 (CCNA1)	1.3	2.3	2.8	NC	1.5	2.5	NC	NC	2.0
L49506.1 cyclin G2	-2.1	2.0	2.6	-2.8	2.3	2.3	NC	3.7	2.5
NM_004354.1 cyclin G2 (CCNG2)	-1.9	3.0	3.2	NC	2.5	2.3	1.0	4.6	3.5
AL535380 B-cell translocation gene 1, anti-proliferative	1.1	1.7	3.5	NC	1.4	2.8	-1.5	1.6	2.6
NM_006472.1 upregulated by 1,25-dihydroxyvitamin D-3 (VDUP1)	1.3	2.1	-2	1.0	2.1	2.3	1.0	2.3	NC
NM_015675.1 growth arrest and DNA-damage-inducible, beta (GADD45B)	1.2	2.5	6.5	1.6	1.5	2.6	1.0	NC	3.5
AF087853.1 growth arrest and DNA damage inducible protein beta	NC	2.0	4.6	1.3	1.2	2.3	NC	NC	3.2
AF078077.1 growth arrest and DNA-damage-inducible protein GADD45beta	1.5	2.0	5.3	1.1	1.2	2.1	NC	1.4	2.8
***Transcription and translation***									
NM_007111.1 transcription factor Dp-1 (TFDP1)	NC	-1.6	-3.2	-1.3	-1.1	-2.0	NC	-1.1	-2.0
NM_012068.2 activating transcription factor 5 (ATF5)	NC	-2.1	-3.5	NC	-1.5	-2.1	-1.3	-1.1	-2.6
NM_012251.1 transcription factor A, mitochondrial (TFAM)	1.3	-1.5	-2.5	NC	-1.6	-2.0	-1.1	-2.0	-3.7
AF220509.1 transcription associated factor TAFII31L	NC	-2.3	-3.2	-1.3	-1.3	-2.3	NC	-1.2	-5.7
AA393940 eukaryotic translation initiation factor 5A	-1.3	-1.5	-3.0	-1.6	-2.0	-3.5	-1.1	-1.1	-2.0
NM_001674.1 activating transcription factor 3 (ATF3)	1.4	1.9	14.9	1.5	2.1	3.5	1.1	2.6	4.9
***Oncogenesis and other***									
NM_001511.1 GRO1 oncogene (GRO1)	2	-1.5	-3.0	1.7	1.2	-2.0	NC	NC	-4.0
NM_002090.1 GRO3 oncogene (GRO3)	2.8	-3.0	-4.0	2.5	-1.4	-6.5	NC	-2.8	-13.9
NM_005754.1 Ras-GTPase-activating protein SH3-domain-binding protein	NC	-1.7	-2.8	NC	-2.0	-2.5	NC	-2.6	-4.6
NM_000026.1 adenylosuccinate lyase (ADSL)	NC	-1.3	-2.5	NC	-1.4	-2.6	NC	-1.6	-3.0
M80261.1 apurinic endonuclease (APE)	-1.1	-1.4	-3.0	-1.1	-1.9	-2.6	NC	-2.1	-3.7
D13413.1 tumor-associated 120 kDa nuclear protein p120	-1.1	-2.0	-3.2	-1.7	-1.7	-2.5	-2.3	-1.1	-3.0
AI743685 methionine aminopeptidase; eIF-2-associated p67	NC	-2.6	-2.0	1.5	-1.5	-3.2	NC	-2.3	-2.8
NM_002634.2 prohibitin (PHB)	NC	-1.4	-3.0	NC	-1.9	-2.1	NC	-1.5	-2.6
NM_002546.1 osteoprotegerin	1.5	-1.3	-3.0	1.2	-2.1	-3.0	NC	-2.8	-5.3
AF003934.1 prostate differentiation factor mRNA	-1.5	4.3	26.0	-1.7	4.9	16.0	NC	4.3	12.1
NM_000177.1 gelsolin (amyloidosis, Finnish type) (GSN)	NC	1.5	2.8	NC	2.0	3.5	NC	1.9	2.3
***Invasion and metastasis***									
NM_003254.1 tissue inhibitor of metalloproteinase 1 (TIMP1)	NC	2.0	2.5	NC	2.6	3.7	NC	2.6	3.7
NM_003255.2 tissue inhibitor of metalloproteina(TIMP2)	NC	1.6	2.5	NC	2.3	3.5	NC	1.6	3.5
NM_002638.1 protease inhibitor 3 (PI3)	1.2	2.0	2.0	1.1	3.7	5.7	1.0	3.0	4.6
NM_005562.1 laminin, gamma 2 (nicein (100 kD)	1.3	1.5	2.0	1.1	2.5	3.7	NC	2.3	3.7
NM_001908.1 cathepsin B (CTSB)	NC	1.7	3.0	NC	1.7	2.8	1.1	1.3	2.5
NM_002658.1 plasminogen activator, urokinase (PLAU)	NC	2.3	3.5	NC	2.8	2.3	1.0	2.1	1.9
NM_000930.1 plasminogen activator, tissue (PLAT)	1.1	2.5	4.6	NC	5.7	9.8	NC	3.2	4.9
NM_002421.2 matrix metalloproteinase 1 (MMP1)	NC	1.4	4.3	NC	4.9	18.4	NC	5.7	13.0
NM_004994.1 matrix metalloproteinase 9 (MMP9)	1.1	1.7	2.0	1.0	1.9	2.0	NC	2.5	2.1
NM_000435.1 Notch homolog 3 (NOTCH3)	NC	2.0	2.6	-2	2.3	3.2	NC	3.5	3.5
***Resistance to chemotherapeutic agents***									
NM_000499.2 cytochrome P450, subfamily I (CYP1A1)	1.4	3.0	4.0	NC	3.5	5.3	1.2	9.2	10.6
NM_006697.1 cisplatin resistance associated (CRA)	1.0	1.7	2.0	-1.3	2.0	2.6	NC	NC	2.6
NM_005980.1 S100 calcium-binding protein P (S100P)	-1.2	4.9	24.3	1.1	10.6	27.9	NC	6.5	19.7
NM_002961.2 S100 calcium-binding protein A4 (S100A4)	3.0	3.7	5.3	1.0	5.7	11.3	2.8	6.5	10.6
NM_020672.1 S100-type calcium binding protein A14 (LOC57402)	NC	1.5	2.0	NC	2.1	2.6	1.1	1.6	2.1
NM_001894.1 casein kinase 1, epsilon (CSNK1E)	NC	1.7	3.2	NC	2.0	2.6	1.1	1.7	3.2
NM_000700.1 annexin A1 (ANXA1)	NC	1.9	2.3	NC	2.0	2.3	NC	1.6	2.1

The genes in this list showed a >2 fold change in expression in at least one time point in both mono and combination treatment.

NC: No change; Negative value: Decrease; Positive value: Increase.

**Table 3:** Fold change of genes in LNCaP cells exposed to combination treatment or mono-treatment with Taxotere or Furtulon

**Gene**	**Taxotere**			**Furtulon**			**Taxotere+Furtulon**		
			
	**6 h**	**36 h**	**72 h**	**6 h**	**36 h**	**72 h**	**6 h**	**36 h**	**72 h**
***Cell cycle and apoptosis***									
NM_001786.1 cell division cycle 2 (CDC2)	-1.2	-7.5	-12.1	-1.1	-2.8	-1.6	NC	-4.0	-14.9
D88357.1 mRNA for CDC2 delta T	-1.1	-6.1	-14.9	NC	-2.8	-1.6	-1.1	-4.3	-13.0
NM_001237.1 cyclin A2 (CCNA2)	-1.2	-5.7	-12.1	NC	NC	-2.1	-1.1	-8.6	-13.9
NM_004702.1 cyclin E2 (CCNE2)	1.6	-5.3	-3.2	NC	-2.3	-1.7	-1.7	-4.9	-8.0
AF112857.1 cyclin E2 splice variant 1 mRNA	1.2	-3.5	-3.2	NC	-2.0	-1.6	-1.4	-7.0	-8.6
NM_001761.1 cyclin F (CCNF)	-1.5	-2.5	-3.7	-1.1	-1.1	-2.0	-1.4	-2.3	-2.8
AB012305.1 cyclin-dependent kinase 2	-1.7	-3.5	-5.3	-2.0	-2.1	-2.1	NC	-2.8	-3.5
U30872.1 mitosin mRNA	-1.2	-4.6	-6.1	NC	-2.1	-1.3	NC	-3.2	-7.0
NM_000389.1 cyclin-dependent kinase inhibitor 1A (p21, Cip1)	1.3	9.8	8.6	1.0	2.0	2.0	1.0	8.0	7.5
NM_006763.1 BTG family, member 2 (BTG2)	1.5	6.1	6.5	1.1	1.7	2.1	1.4	5.3	3.7
NM_006472.1 upregulated by 1,25-dihydroxyvitamin D-3 (VDUP1)	1.0	3.5	3.5	1.0	1.5	2.6	1.0	2.3	3.0
NM_001924.2 growth arrest and DNA-damage-inducible, alpha	1.4	7.5	7.5	NC	1.9	2.6	1.1	7.0	7.5
BC003637.1 Similar to DNA-damage-inducible transcript 3	1.0	2.6	2.6	1.1	2.1	2.5	NC	2.6	2.8
NM_014454.1 p53 regulated PA26 nuclear protein (PA26)	1.1	4.0	3.0	1.1	1.4	2.6	1.1	3.7	3.7
NM_004701.2 cyclin B2 (CCNB2)	-1.1	-8	-13.9	NC	1.3	-1.3	-1	-4.9	-19.7
NM_001255.1 CDC20 (cell division cycle 20)	-1.9	-90.5	-147	NC	1.6	-1.9	-1.6	-13	-157
NM_021873.1 cell division cycle 25B (CDC25B)	-1.1	-1.6	-2.5	NC	NC	-1.4	-1	-2.3	-3.25
NM_001827.1 CDC28 protein kinase 2 (CKS2)	-1.1	-4.6	-6.5	NC	1.6	-1.6	-1.2	-3.5	-5.7
NM_001168.1 survivin (BIRC5)	-1.1	-27.9	-294	NC	NC	-1.6	NC	-6.9	-181
***Transcription, translation, oncogenesis, angiogenesis, other***									
NM_021953.1 forkhead box M1 (FOXM1)	-1.2	-13.9	-42.2	-1.1	-1.2	-2.0	NC	-2.8	-45.3
NM_000465.1 BRCA1 associated RING domain 1 (BARD1)	-1.3	-1.9	-2.0	NC	-1.1	-2.1	-1.2	-2.6	-4.9
NM_003368.1 ubiquitin specific protease 1 (USP1)	NC	-2.6	-1.6	NC	-2.0	-1.4	NC	-2.1	-1.9
NM_006716.1 activator of S phase kinase (ASK)	-1.2	-6.5	-5.3	-1.2	-1.3	-2.0	-1.1	-4.6	-11.3
NM_003246.1 thrombospondin 1 (THBS1)	NC	-2.0	-3.5	-1.9	-1.4	-2.6	-1.1	-1.6	-4.6
NM_001147.1 angiopoietin 2 (ANGPT2)	3.5	3.5	8.0	1.7	5.3	8.0	-0.5	4.9	2.8
AF187858.1 angiopoietin-2 isoform-1	NC	2.6	4.3	NC	1.5	3.5	NC	2.0	2.0
NM_000435.1 Notch (Drosophila) homolog 3 (NOTCH3)	1.3	3.0	2.8	1.0	2.3	2.1	NC	2.1	3.0
NM_001674.1 activating transcription factor 3 (ATF3)	NC	1.5	2.5	NC	1.7	2.5	NC	2.6	4.3
NM_003158.1 serinethreonine kinase 6 (STK6)	-1.7	-11.3	-9.8	1.1	1.2	-1.7	-1.3	-8.6	-26
NM_003600.1 serinethreonine kinase 15 (STK15)	-1.2	-3.7	-8.6	NC	NC	-1.6	-1.3	-4	-6.1
AF162704.1 androgen receptor mRNA	NC	1.3	-1.6	1.2	1.3	-2.1	-1.1	-1.6	-2
***Resistance to chemotherapeutic agents***									
NM_000693.1 aldehyde dehydrogenase 1A3 (ALDH1A3)	NC	4.0	3.2	1.0	1.1	2.1	1.2	4.9	4.3
NM_005980.1 S100 calcium-binding protein P (S100P)	1.0	5.7	4.0	1.5	2.6	3.0	NC	2.6	2.6

The genes in this list showed a >2 fold change in expression in at least one time point in both mono and combination treatment.

NC: No change; Negative value: Decrease; Positive value: Increase.

**Table 4:** Comparison of difference in gene expression between combination treatment and mono-treatment in PC3 cells

**Gene**	**Taxotere+Furtulon**		
	
	**6 h**	**36 h**	**72 h**
***Decrease in combination treatment, No change or increase in mono-treatment.***			
NM_001261.1 cyclin-dependent kinase 9 (CDC2-related kinase) (CDK9)	-1.3	-1.2	-2.3
NM_016507.1 CDC2-related protein kinase 7 (CrkRS)	NC	-1.6	-2.3
AF080157.1 IkB kinase-a (IKK-alpha)	NC	-2.0	-1.6
U62296.1 transcription factor NF-YC	-1.1	-1.7	-2.3
BC005003.1 nuclear transcription factor Y, gamma	-1.1	-2.0	-2.1
BC001771.1 transcription factor IIF	NC	-1.6	-2.3
AI434345 activating transcription factor 1	NC	-2.5	-2.6
BC001173.1 eukaryotic translation initiation factor 3	NC	-1.3	-2.0
U78525.1 eukaryotic translation initiation factor (eIF3)	NC	-1.4	-2.0
NM_001814.1 cathepsin C (CTSC)	NC	-1.3	-2.0
NM_003377.1 vascular endothelial growth factor B (VEGFB)	-2.1	NC	NC
AF035620.1 BRCA1-associated protein 2 (BRAP2)	-2.3	NC	NC
AF035620.1 BRCA1-associated protein 2 (BRAP2)	-2.3	-2.0	-1.4
NM_005346.2 heat shock 70 kD protein 1B (HSPA1B)	-1.1	-2.1	-1.1
BC000478.1 heat shock 70 kD protein 9B	NC	-1.7	-2.3
NM_014278.1 heat shock protein (hsp110 family) (APG-1)	-1.2	-1.3	-2.0
BC002526.1 Similar to heat shock protein, 110 kDa	-1.1	-1.4	-2.1
***Increase in combination treatment, No change or decrease in mono-treatment.***			
NM_000077.1 cyclin-dependent kinase inhibitor 2A (p16, inhibits CDK4)	NC	1.7	2.1
NM_001262.1 cyclin-dependent kinase inhibitor 2C (p18, inhibits CDK4)	NC	2.1	2.0
J03202.1 laminin B2 chain mRNA	NC	1.3	2.0
BG164365 microtubule-associated protein 1B	NC	2.3	2.0
NM_000594.1 tumor necrosis factor, member 2	NC	2.5	1.6
NM_001065.1 tumor necrosis factor, member 1A	NC	2.0	1.9
BC000125.1 Similar to transforming growth factor, beta 1	-1.2	1.9	2.1
NM_005649.1 transcription factor 17 (TCF17)	NC	1.1	2.3
NM_005923.2 mitogen-activated protein kinase kinase kinase 5 (MAP3K5)	NC	1.6	2.1
NM_005204.1 mitogen-activated protein kinase kinase kinase 8 (MAP3K8)	NC	2.3	4.3
NM_000785.1 cytochrome P450, subfamily XXVIIB	NC	2.1	1.5
NM_002960.1 S100 calcium-binding protein A3 (S100A3)	NC	2.5	2.5
NM_002962.1 S100 calcium-binding protein A5	NC	8.6	10.6

NC: No change; Negative value: Decrease; Positive value: Increase.

The genes in this list showed a >2 fold change in expression in at least one time point in combination treatment.

**Table 5:** Comparison of difference in gene expression between combination treatment and mono-treatment in LNCaP cells

**Gene**	**Taxotere+Furtulon**		
	
	**6 h**	**36 h**	**72 h**
***Decrease in combination treatment, No change or increase in mono-treatment.***			
NM_001256.1 cell division cycle 27 (CDC27)	NC	-1.6	-2.0
NM_001963.2 epidermal growth factor (beta-urogastrone) (EGF)	NC	-3.5	-1.9
NM_004113.2 fibroblast growth factor 12B (FGF12B)	NC	-4.3	-1.2
U13022 negative regulator of programmed cell death ICH-1S (Ich-1)	NC	-2.3	-2.0
AF249273.1 Bcl-2-associated transcription factor short form mRNA	NC	-1.7	-2.3
NM_001071.1 thymidylate synthetase (TYMS)	NC	-1.4	-2.6
AF005068.1 breast and ovarian cancer susceptibility protein (BRCA1)	NC	-4.6	-17.1
NM_012068.2 activating transcription factor 5 (ATF5)	NC	-2.0	-2.8
NM_021809.1 TGF(beta)-induced transcription factor 2 (TGIF2)	NC	-1.6	-2.1
NM_001412.1 eukaryotic translation initiation factor 1A (EIF1A)	NC	-1.2	-2.3
NM_002758.1 mitogen-activated protein kinase kinase 6 (MAP2K6)	NC	-1.7	-2.1
***Increase in combination treatment, No change or decrease in mono-treatment.***			
NM_006034.1 p53-induced protein (PIG11)	NC	6.1	7.5
NM_000227.1 laminin, alpha 3	NC	1.7	2.0
NM_000094.1 collagen, type VII, alpha 1 (COL7A1)	NC	1.4	6.5
NM_016437.1 tubulin, gamma 2 (TUBG2)	NC	1.4	2.1
NM_000853.1 glutathione S-transferase theta 1 (GSTT1)	NC	2.0	2.1

NC: No change; Negative value: Decrease; Positive value: Increase.

The genes in this list showed a >2 fold change in expression in at least one time point in combination treatment.
